# Respiratory Gymnastic Methods

**Published:** 1902-07

**Authors:** 


### Respiratory Gymnastic Methods.

Under the title of "Respiratory Gymnastic Methods," A. Abrams has a valuable article in *American Medicine*. The principal methods are:

First. Stimulation by means of shock to the peripheral nerves, as applied by cold water and friction, the peripheral nerves having more control over the respiration than the vagus branches.

Second. Forced breathing, which is more valuable than any complicated system of lung development.

Third. Changing the type of breathing from costal to abdominal and vice versa often gives most satisfactory results. An elastic band to the upper chest causes the type to become abdominal, while if the chest expansion is unsatisfactory this band to the upper abdominal region will cause a fuller expansion of the upper chest.

Continuing, the author cites the advantage of treating various pulmonary diseases, especially phthsis, by these means, as fresh air forced into the uttermost parts of a diseased lung has proven to be of the greatest importance as regards a cure; not only is the detritus raised with greater ease, but a healthy reaction is set up in the diseased area. C. R. M.

